# MED15 overexpression in prostate cancer arises during androgen deprivation therapy via PI3K/mTOR signaling

**DOI:** 10.18632/oncotarget.13860

**Published:** 2016-12-10

**Authors:** Anne Offermann, Ignacija Vlasic, Isabella Syring, Wenzel Vogel, Christian Ruiz, Tobias Zellweger, Cyrill A Rentsch, Susanne Hagedorn, Jochen Behrends, Michael Nowak, Axel Merseburger, Lukas Bubendorf, Jutta Kirfel, Stefan Duensing, David Adler, Sven Perner

**Affiliations:** ^1^ Pathology of the University Medical Center Schleswig-Holstein, Campus Luebeck and Research Center Borstel, Leibniz Center for Medicine and Biosciences, Borstel, Germany; ^2^ Department of Urology, University Hospital Bonn, Bonn, Germany; ^3^ Institute for Pathology, University Hospital Basel, Basel, Switzerland; ^4^ Department of Urology, St. Claraspital, Basel, Switzerland; ^5^ Department of Urology, University Hospital Basel, Basel, Switzerland; ^6^ Section of Prostate Cancer Research, Center for Integrated Oncology Cologne/Bonn, University Hospital of Bonn, Bonn, Germany; ^7^ Institute of Pathology, Center for Integrated Oncology Cologne/Bonn, University Hospital of Bonn, Bonn, Germany; ^8^ Core Facility Fluorescence Cytometry, Research Center Borstel, Leibniz Center for Medicine and Biosciences, Borstel, Germany; ^9^ Department of Urology, University Hospital Luebeck, Luebeck, Germany; ^10^ Section of Molecular Urooncology, Department of Urology, University of Heidelberg School of Medicine, Heidelberg, Germany

**Keywords:** mediator complex, MED15, castration-resistant prostate cancer, androgen deprivation, PI3 kinase

## Abstract

Androgen deprivation therapy (ADT) is the main therapeutic option for advanced prostate cancer (PCa). After initial regression, most tumors develop into castration-resistant PCa (CRPC). Previously, we found the Mediator complex subunit MED15 to be overexpressed in CRPC and to correlate with clinical outcome. Therefore, we investigated whether MED15 is implicated in the signaling changes taking place during progression to CRPC. Immunohistochemistry (IHC) for MED15 on matched samples from the same patients before and after ADT reveals significantly increased MED15 expression after ADT in 72%. A validation cohort comprising samples before and after therapy confirmed our observations. Protein analysis for pAKT and pSMAD3 shows that MED15 correlates with PI3K and TGFß activities, respectively, and that hyper-activation of both pathways simultaneously correlates with highest levels of MED15. We further show that MED15 protein expression increases in LNCaP cells under androgen deprivation, and via EGF mediated PI3K activation. PI3K/mTOR and TGFß-receptor inhibition results in decreased MED15 expression. *MED15* knockdown reduces LNCaP cell viability and induces apoptosis during androgen deprivation, while cell cycle is not affected. Collectively, MED15 overexpression arises during ADT via hyper-activation of PI3K/mTOR signaling, thus MED15 may serve as a predictive marker for response to PI3K/mTOR inhibitors. Furthermore, MED15 is potentially a therapeutic target for the treatment of CRPC.

## INTRODUCTION

Prostate Cancer (PCa) is a clinically heterogeneous disease with > 900,000 diagnoses annually, and remains the third most common cause of cancer associated death in developed countries [1]. Early stage PCa depends on the androgen-receptor (AR) as a major mediator of growth and survival, and is driven by androgens [2]. Therefore, recurrent disease after failure of localized treatments and metastatic tumors are systematically treated with androgen deprivation therapy (ADT), which initially leads to tumor growth arrest and disease regression [3, 4]. However, most of these tumors unfortunately develop into castration-resistant prostate cancer (CRPC) despite the low levels of androgens, leading to a median survival rate of 2-3 years from the time of initiation of ADT [5]. The identification of prognostic markers that distinguish the aggressive from the indolent form of PCa is required to select patients for different treatment options [6]. Furthermore, unraveling the mechanisms that underlie the pathogenesis of castration-resistance provide the basis for the development of novel therapeutic targets [7]. Therefore, many studies examined the molecular differences between hormone-sensitive PCa and castration-resistant PCa after ADT [8–10], and describe how PCa cells are able to escape their initial androgen dependence [7, 11].

We previously found MED15, a subunit of the multi-protein complex Mediator, to be overexpressed at much higher frequency in CRPC than in primary PCa, and to correlate with worse clinical outcome [12]. The Mediator complex is a key regulator of the transcription of protein-coding genes [13] and serves as an integrative hub for diverse signaling pathways [14]. The Mediator can be divided into four distinct modules: the head, middle, tail and kinase, and is comprised of 30 subunits in humans [14]. Several studies reported Mediator subunits to be involved in human cancers [15] and our group analyzed the transcriptional profile of all subunits across different cancer types [16]. Notably, the tail subunit MED15 interacts with different co-activators, and is required for TGFß signaling [17–19]. Previously, we observed MED15 to be implicated in TGFß signaling in PCa [12], a growth-promoting pathway in advanced PCa [20, 21] which is critical for CRPC progression [21]. Interestingly, TGFß mediated phosphatidylinositol 3-kinase (PI3K) activation during cancer progression leads to pro-oncogenic signaling promoting invasion [22], proliferation and survival [23] of cancer cells. At the same time, the PI3K signaling is commonly altered in CRPC [24], up-regulated in response to androgen ablation [25], and required for the maintenance of castration-resistant growth as well as survival of PCa cells [26]. Therefore, efforts have been made to develop therapeutic agents targeting the TGFß or PI3K signaling cascade in patients with CRPC [27, 28]. However, despite antitumor activity in preclinical models [27, 28], ongoing challenges remain and include unraveling the resistance mechanisms, identification of biomarkers that predict drug responsiveness, and generating new therapeutic targets which might enhance and specify TGFß and PI3K signaling inhibition [28].

Our previous findings showing that MED15 is overexpressed at high frequency in CRPC and is implicated in TGFß signaling [12], prompted us to investigate whether MED15 is involved in the signaling network changes observed during the progression to CRPC which is critically driven by ADT [7]. Furthermore, we aimed to explore whether MED15 is required for PCa cell survival under androgen deprived conditions.

## RESULTS

### Increased MED15 expression in response to androgen deprivation therapy in PCa tissue

**Figure 1:**
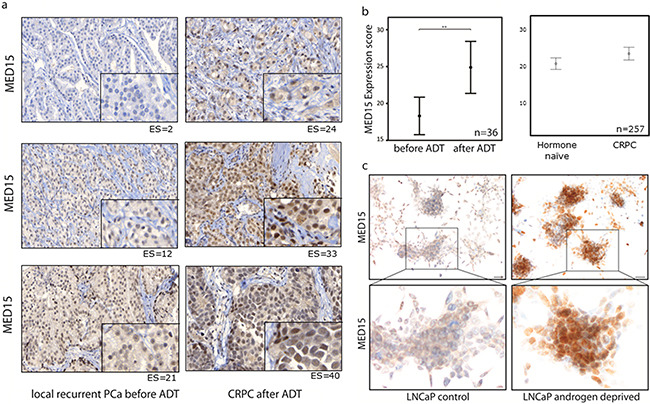
Increased MED15 expression following androgen deprivation of PCa cells a. Increased MED15 expression by immunohistochemistry in local recurrent CRPC after ADT (right) compared to hormone-naïve local recurrent PCa from same patient before ADT (left). Expression score for each staining is mentioned below. Scale bar, 50µm or 10µm. b. Higher mean expression score (ES) of MED15 in 36 local recurrent CRPC samples after ADT (ES=24.9) compared to 29 local recurrent PCa samples before ADT (ES=18.3) of same patients. Higher mean ES of MED15 in 145 CRPC samples (23,9) compared to 112 hormone-naïve PCa samples (20,9). Bars indicate the mean with 95% coincidence interval of the MED15 ES (independent t-test, ** p < 0.01). Data are represented as mean ± SEM. c. Increased MED15 expression in LNCaP cells grown under androgen deprived conditions compared to cells grown in the presence of androgens as control after 72h by immunocytochemistry. Scale bar 50µm.

In order to investigate whether the increased expression of MED15 is a response to androgen deprivation, we performed immunohistochemistry (IHC) for MED15 on hormone-naïve local recurrent PCa tissue before and local recurrent castration-resistant PCa after androgen deprivation therapy (ADT) from the same patients. Out of 29 patients, 21 patients (72%) harbored increased MED15 expression levels in PCa tissue after ADT compared to levels in tissues before undergoing ADT (Figure 1a). Analysis of the MED15 expression before and after ADT from same patients revealed MED15 to be significantly up-regulated in response to ADT (paired sample t-test, *** p < 0.001). Further, the mean MED15 expression score (ES) in CRPC tissue after ADT (ES=24.9) was significantly higher compared to the mean MED15 expression in hormone-naïve tissue before therapy (ES=18.3) (Figure 1b).

To support our results described above, we performed IHC for MED15 on a validation cohort comprising 112 hormone-naïve PCa tissues from untreated patients as well as 145 castration-resistant PCa tissues from patients who underwent ADT. We observed a higher mean ES for MED15 in CRPC tissues after ADT (ES=23.9) compared to hormone-naïve PCa tissues from untreated patients (ES=20.9) (independent t-test, * p < 0.05) (Figure 1b).

### Increased MED15 expression in response to androgen deprivation in PCa cell lines

To mimic the tissue findings *in vitro*, we used androgen-sensitive LNCaP cells as a cell line model to investigate the effect of androgen deprivation upon MED15 expression. Therefore, cells were grown in medium containing charcoal-stripped serum (CS FBS) with decreased levels of hormones without loss of other serum components, or normal serum (FBS) with physiological doses of androgens (1-10 nM) as control. We observed a significantly higher MED15 protein expression in cells grown under androgen deprived conditions compared to cells grown in the presence of androgens analyzed by immunocytochemistry (ICC) (Figure 1c) and western blot (Figure 4d). We further analyzed transcriptional MED15 expression by qRT-PCR and found no significant difference of MED15 levels between cells grown in the presence or absence of androgens (Supplementary Figure 2b). To rule out unspecific effects under AD, we included the CRPC cell line PC3 into experiments. We could not observe different MED15 levels in PC3 cells grown under normal conditions or AD (Figure 4d).

**Figure 3:**
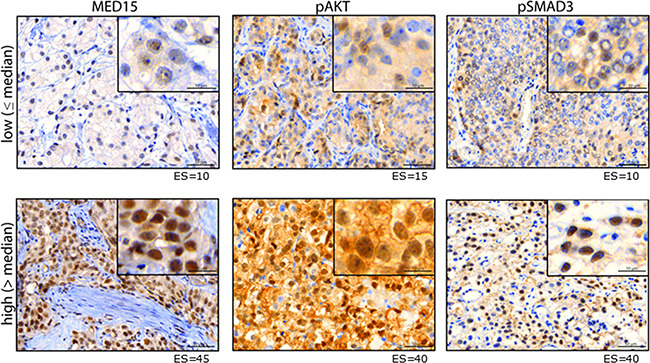
MED15 up-regulation after androgen deprivation correlates with PI3K and TGFß signaling a. Higher mean ES of MED15 in samples with high pAKT and b. high pSMAD3 expression in unmatched PCa samples compared to samples with low pAKT and low pSMAD3 expression. Bars indicate the mean with 95% coincidence interval of the MED15 ES n= 236 samples and n=227 samples for pAKT and pSMAD3 evaluation, respectively (independent t-test, ** p < 0.01). c. Increased MED15 expression after ADT in samples with high pAKT or pSMAD3 staining and highest MED15 expression in samples showing simultaneous high pAKT and pSMAD3 staining. Bars indicate the mean with 95% coincidence interval of the MED15 ES. n= 127 samples (independent t-test, * p < 0.05, *** p < 0.001). d. MED15 expression following ADT in matched tissues from same patients is increased expressed in samples showing an up-regulation of pAKT signaling in CRPC tissue after ADT when compared to samples showing unchanged pAKT staining before and after ADT (independent t-test, * p < 0.05). Expression score for each staining is mentioned below. Data are represented as mean ± SEM.

### MED15 expression correlates with PI3K and TGFß activity in PCa tissues

**Figure 2:**
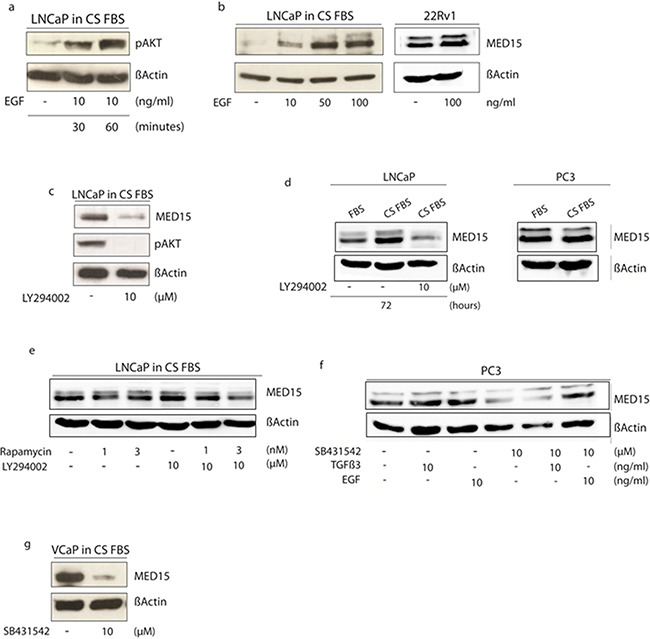
Protein expression of MED15, pAKT and pSMAD3 Representative images of PCa tissue with low (upper panel) or high (lower panel) immunhistochemical staining for MED15, pAKT and pSMAD3. Expression score for each staining is mentioned below. Scale bar, 50µm or 10µm.

IHC for pAKT and pSMAD3 was performed on PCa tissues to analyze the activity of PI3K and TGFß signaling, respectively (Figure 2). In matched tissues before and after ADT from same patients, we observed that MED15 correlates significantly with the expression of pAKT in both, tissues before and after ADT (Pearson correlation, p < 0.05). In support of this observation, the ES of MED15 correlated with pAKT as well as pSMAD3 expression in our validation cohort comprising PCa tissues from unmatched patients (Pearson correlation, p < 0.01). Consistently, we found that tissue samples with high pAKT and pSMAD3 staining harbored increased MED15 expression compared to samples with low pAKT and pSMAD3 expression levels (Figure 3a, 3b). MED15 was expressed at the highest levels in CRPC tissues after ADT in samples with simultaneous high pAKT and pSMAD3 stainings (Figure 3c).

**Figure 4:**
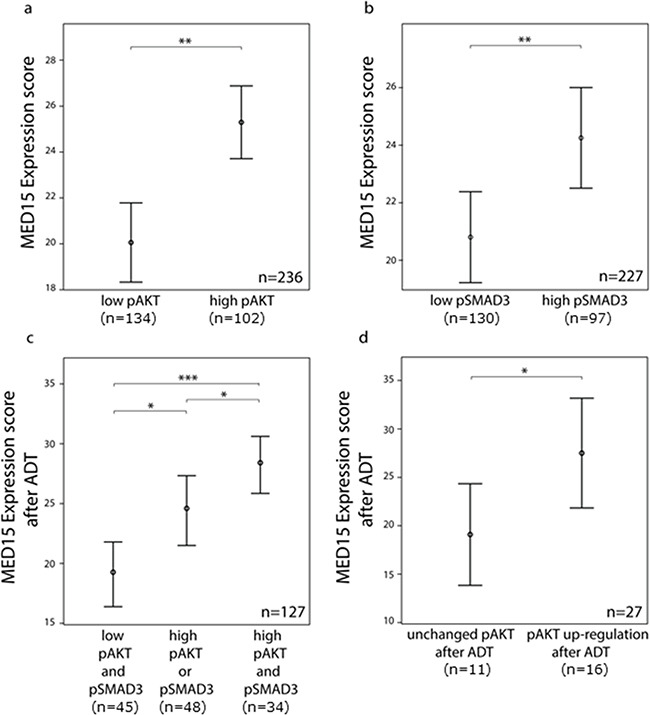
Modulation of PI3K and TGFß signaling in cell lines affects MED15 up-regulation following androgen deprivation of cells a. Phosphorylation of AKT in response to EGF mediated PI3K signaling activation by western blot analysis. b. LNCaP cells grown in medium containing charcoal-stripped fetal bovine serum (CS FBS) show increased MED15 expression upon EGF treatment in a dose-dependent manner. 22Rv1 cells grown under physiological conditions show slight increased MED15 expression upon EGF treatment. c. Treatment with 10µM PI3K inhibitor LY294002 results in reduced pAKT and MED15 expression. d. LNCaP cells in medium without androgens (CS FBS) show higher MED15 expression compared to cells grown in the presence of 1-10 nM androgens (FBS). This effect was abolished by PI3K inhibition using 10µM LY294002 under same conditions. PC3 cells growing in CS FBS showed no different MED15 expression compared to control cells (FBS). e. mTOR inhibition by rapamycin (1 and 3nM) reduces MED15 expression in LNCaP cells grown under androgen deprived conditions only slightly. Combined PI3K and mTOR inhibition by LY294002 (10µM) and rapamycin (1 and 3nM) treatment under same conditions leads to reduced MED15 expression. f. PC3 cells grown in full-growth medium were treated with EGF, TGFß, TGFß-receptor inhibitor SB431542 alone, or in combination with SB431542 and TGFß or SB431542 and EGF. Reduced MED15 expression in SB431542 treated cells and no expression changes in response to exogenous TGFß3. EGF treatment leads to increased MED15 expression in the presence of SB431542. g. SB431542 treatment of VCaP cells grown under androgen deprived conditions (CS FBS) results in decreased MED15 expression.

### PI3K activation after androgen deprivation correlates with higher MED15 expression

To investigate whether increased MED15 expression after ADT may be affected by PI3K hyper-activation, we first compared the MED15 expression between PCa samples with or without increased pAKT staining after ADT compared to the matched tissues prior to treatment. Patients that harbored higher levels of pAKT in CRPC tissue after ADT compared to matched hormone-naïve samples prior to therapy (fold change > 1.1) exhibited a significantly higher MED15 expression following ADT compared to samples without increased pAKT (Figure 3d). Furthermore, out of the 8 patients who did not exhibit an increase in MED15 expression after ADT, 6 patients exhibited no increase in pAKT staining after ADT as well.

### PI3K activation during androgen deprivation leads to increased MED15 expression in PCa cells

LNCaP cells are androgen-sensitive human PCa cells derived from lymph node metastasis and thus can serve as an *in vitro* model for the investigation of the development of castration resistance. We activated the PI3K signaling by treating androgen-sensitive LNCaP cells with recombinant epidermal growth factor (EGF) during androgen deprived conditions. Phosphorylation of AKT in response to EGF treatment confirms the activation of the PI3K pathway (Figure 4a). Western blot analysis showed increased expression of MED15 in a dose-dependent manner after 24 hours of EGF treatment (Figure 4b). PI3K inhibition using 10µM LY294002 was sufficient to prevent the phosphorylation of AKT (Figure 4c) and to reduce MED15 on protein level in LNCaP cells under androgen deprived conditions after 24 hours (Figure 4c). LNCaP cells were then grown in the presence (FBS) or absence (CS FBS) of androgens with or without the PI3K inhibitor LY294002 (Figure 4d). Western blot showed increased MED15 expression in LNCaP cells grown under androgen deprived conditions for 72 hours, which was abolished by PI3K inhibition by LY294002 treatment under same conditions (Figure 4d). We further inhibited mTOR, a downstream molecule of PI3K/AKT signaling, by treating cells with 1 or 3nM rapamycin alone, with PI3K inhibitor LY294002 alone or combined treatments and found that MED15 decreases only slightly with low rapamycin doses alone, but was reduced significantly even after 24 hours when PI3K and mTOR inhibitors were combined (Figure 4e). In PTEN wild-type cells, EGF treatment with 100 ng/ml for 24 hours only slightly increased MED15 protein expression (Figure 4b).

### TGFß signaling inhibition reduces MED15 expression

As described in our previously study [12], TGFß3 treatment after serum starvation leads to increased MED15 expression in PC3 cells. To investigate whether inhibition of TGFß signaling reduces MED15 expression, we treated TGFß-receptor positive PC3 cells with the TGFß-receptor blocker SB431542. We found decreased MED15 expression in response to SB431542 after 24 hours by western blot analysis (Figure 4f). The blocked TGFß-receptor prevents MED15 up-regulation in response to exogenous TGFß3 (Figure 4f), while EGF leads to increased MED15 expression despite TGFß-receptor inhibition (Figure 4f). TGFß3 or EGF without serum starvation does not increase MED15 levels when cell are growing under physiological conditions (Figure 4f). To inhibit TGFß signaling under androgen deprived conditions, we treated androgen-dependent and TGFß-receptor positive VCaP cells with SB431542 grown in charcoal stripped medium (Figure 4g). We found reduced MED15 expression in SB431542 treated VCaP cells compared to untreated cells (Figure 4g).

### *MED15* knockdown reduces cell survival under androgen deprivation and effects expression of genes of the AR signaling axis

**Figure 5:**
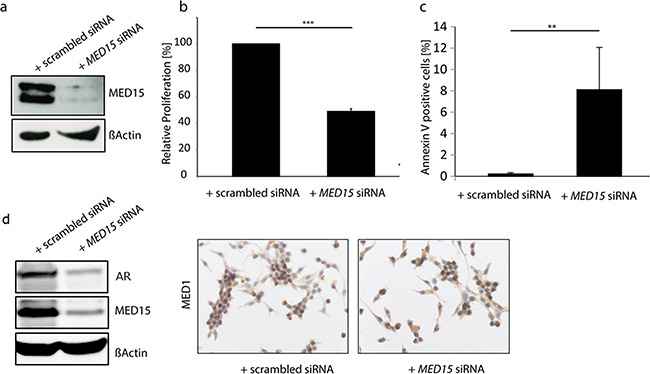
MED15 knockdown reduces cell viability and induces apoptosis in LNCaP cells under androgen deprived conditions a. Reduced MED15 expression in LNCaP cell which were treated with MED15 specific siRNA shown by western blot. b. MTT assay reveals 50% reduction of LNCaP cell viability after MED15 knockdown when cells were grown under androgen deprivation for 72 hours. Absorbance at 595nm was normalized to control cells (one-sample t-test, *** p < 0.001). Data are represented as mean ± SEM. c. Percentage of Annexin V-positive LNCaP cells which were treated with scrambled or MED15 specific siRNA under androgen deprivation (independent t-test, ** p < 0.01). Data are represented as mean ± SEM. d. Reduced AR expression in MED15 knockdown cells compared to control cells shown by western blot. Similar MED1 levels in control and MED15 knockdown cells.

Our results showing that MED15 is up-regulated in response to androgen deprivation and implicated in the PI3K survival pathway prompted us to investigate whether MED15 inhibition affects cell viability under these conditions. Therefore, we performed siRNA mediated *MED15* knockdown in LNCaP cells (Figure 5a) followed by androgen deprivation for 72 hours. Knockdown of *MED15* led to significant reduction of cell viability which was measured by MTT assay (Figure 5b) as well as induction of apoptosis compared to control cells (Figure 5c). There was no differences in cell cycle between control and MED15 knockdown cells (Supplementary Figure 2a). To further investigate effects of *MED15* knockdown on the expression of the AR and its co-regulator MED1, we analyzed AR and MED1 levels on protein level. We found significantly reduced AR expression in LNCaP cells with *MED15* knockdown by western blot (Figure 5d). In contrast, MED1 levels did not change after *MED15* knockdown (Figure 5d).

## DISCUSSION

ADT is the standard therapy option for advanced and metastatic PCa and leads to initial regression of androgen-dependent tumors [2]. However, the majority of patients develops CRPC characterized by androgen-independent tumor progression [7] and poor survival [1]. Molecular profiling of PCa following ADT provides the basis to unravel the mechanisms driving CRPC as well as to identify predictive and novel therapeutic markers. Therefore, several studies identified genes differentially expressed during progression to CRPC [8–10, 29], which are mostly involved in androgen-receptor (AR) or alternative survival pathways [11]. The Mediator complex integrates pathway activation and specific gene expression, and recent studies identified distinct subunits of the Mediator to be involved in different cancer types, including PCa [15, 16]. In 2007, Vijayvargia et al observed a crucial role of the Mediator subunits MED1 and MED17 in PCa [29] and suggested MED1 and MED17 as potential therapeutic targets. Different studies unraveled the molecular mechanisms how MED1 affects AR signaling and malignant properties of PCa cells [30, 31]. In our previous study, we found the Mediator subunit MED15 to be overexpressed at high frequency during PCa progression to CRPC [12]. Additionally, our results showed that MED15 is implicated in TGFß signaling, a pathway which is involved in the progression of CRPC [22] and directly linked to PI3K signaling [32]. Based on our previous findings and due to reports showing that PI3K signaling is up-regulated in response to ADT [25] and serves as a critical survival pathway in CRPC [26], we hypothesized that MED15 overexpression may arise in response to androgen deprivation via the PI3K pathway. Interestingly, we found MED15 to be significantly higher expressed in CRPC tissues after ADT compared to matched hormone-naïve tissues from the same patients before therapy (Figure 1a, 1b). It is worth noting that ADT may provide a selective pressure for cells which are able to survive and grow despite low levels of androgens [2, 33].

In order to investigate whether MED15 up-regulation following ADT may be affected by PI3K activity, we investigated the correlation between them and observed that MED15 correlates significantly with PI3K activity in PCa tissues, and that PCa samples expressing high levels of pAKT harbored increased MED15 expression (Figure 2a, 2b, 3a). Furthermore, MED15 correlates with pAKT expression in tissues before as well as after ADT. In addition, we found MED15 in matched tissues from same patients to be higher expressed in samples with an up-regulation of PI3K signaling in response to therapy (Figure 3d). In contrast, we observed no significant correlation between MED15 and pSMAD3 expression in matched tissues before and after ADT, which may be due to the low sample numbers for pSMAD3 for matched tissues (before ADT n=13, after ADT n=22). However, in our validation cohort (n=227) MED15 correlated significantly with pSMAD3 expression (Figure 3b). Interestingly, several studies described a molecular cross-talk between PI3K and TGFß signaling which may synergistically promote malignant properties in advanced cancer [22, 32]. While PI3K pathway activation can antagonize TGFß induced tumor suppression [34], TGFß has been shown to activate oncogenic PI3K signaling dependent or independent of SMAD signaling [22, 35]. Collectively,our results show increased MED15 expression following ADT, as well as a significant correlation with TGFß and PI3K activity in PCa tissues. These observations prompted us to compare MED15 expression after ADT between tissue samples with low activity of TGFß and PI3K signaling, high activity of one pathway or hyper-activation of both pathways. Interestingly, we observed the highest expression of MED15 after ADT in samples harboring simultaneously high TGFß and high PI3K signaling activation (Figure 3c). Together with the reports that components of both signaling cascades exhibit an increased expression in response to androgen deprivation [36, 25], our results provide evidence that the interplay between these two pathways described previously [32] may lead to increased expression of MED15 in response to androgen deprivation (Supplementary Figure 1 [41]).

In support of our tissue findings, we first used the androgen-dependent LNCaP cells as cell model for investigating changes during androgen deprivation to mimic the tissue conditions *in vitro*. We found that the protein expression of MED15 increased significantly when cells were grown in the absence of androgens (Figure 1c, 4d). In contrast, in the CRPC cell lines PC3 we did not observe changed MED15 levels in response to androgen deprivation (Figure 4d). Based on our results, MED15 up-regulation might be rather mediated by protein stabilization and reduced protein degradation than by direct transcriptional regulation (Supplementary Figure 2b). Interestingly, the effect seen in LNCaP cells could be abolished when cells were treated with the PI3K inhibitor LY294002 under same conditions (Figure 4d). PI3K inhibition for 72 hours reduced significantly the expression of MED15 when cells were grown under androgen deprived conditions (Figure 4c), whereas this effect could not been observed when cells were grown in medium containing physiological doses of androgens (data not shown). Collectively, our results provide evidence that hyper-activation of PI3K signaling leads to the up-regulation of MED15 during androgen deprivation. We found MED15 to be higher expressed after androgen deprivation in tissue samples showing an up-regulation of pAKT after ADT compared to tissues before therapy from same patients. Consistent with this observation, blocking the PI3K abolished the up-regulation of MED15 in LNCaP cells when androgens were removed (Figure 4d), while hyper-activation of PI3K signaling during androgen deprivation increased MED15 expression (Figure 4b). In LNCaP cells, PTEN is inactivated leading to constitutive PI3K activity [37]. To investigate effects of EGF in cells with low PI3K basal activity, we used PTEN-wild type 22Rv1 [37] cells for EGF experiments. Here, we observed only slightly increased MED15 expression in response to EGF treatment after 24 hours (Figure 4b). In 22Rv1 cells, EGF mediated PI3K stimulation might be at least partly reduced by full PTEN activity, which might explain the slight effect upon MED15 expression. Thus, studies aiming to investigate of PI3K functions in PTEN wild type cells have to be optimized by including PTEN inhibitors or *PTEN* knockdown.

Dual PI3K and mTOR inhibitors have been suggested to exhibit more potent antitumor activity compared to agents targeting single components of the PI3K signaling [38]. Furthermore, as activated mTOR results in a signaling cascade which down-regulates PI3K/AKT activity, single mTOR inhibition leads to loss of this feedback loop enhancing PI3K/AKT signaling [38]. Interestingly, we observed MED15 to be reduced only slightly when LNCaP cells were treated with 1 or 3nM mTOR inhibitor rapamycin alone. In contrast, the dual inhibition of both PI3K and mTOR reduced MED15 expression even after 24 hours (Figure 4e). The effect on MED15 reduction differed between treatments for 24 and 72 hours, which might be due to protein half time of MED15 or prolonged effect of PI3K inhibition upon MED15. It has to be considered that there are different downstream pathways from PI3K/AKT signaling in addition to mTOR activation. Further experiments are needed to explore whether the inhibition of other PI3K/AKT downstream components results in decreased MED15 expression, and abolish MED15 up-regulation following androgen deprivation.

As described above, TGFß can lead to direct activation of the PI3K signaling in PCa cells [22]. Notably, recent studies reported that the TGFß 3 increased the metastatic potential of PCa cells in a PI3K and SMAD dependent manner more effectively than other TGFß isoforms [22]. Additionally, TGFß3 was overexpressed in an androgen-independent derivate of LNCaP cells compared to its androgen-dependent parental cell line [36]. Here, our results show that MED15 is expressed at the highest level after ADT when PI3K as well as TGFß signaling are hyper-activated in CRPC tissues (Figure 3c). To investigate whether inhibition of TGFß signaling during androgen deprivation reduces MED15 expression similar to PI3K inhibition, we blocked the TGFß receptor by treating PC3 and VCaP cells with SB431542; LNCaP cells are TGFß-receptor negative [22]. We observed that MED15 expression was decreased in SB431542 treated cells (Figure 4f, 4g), and that this inhibition prevented TGFß3 induced MED15 expression (Figure 4f). Interestingly, EGF induced PI3K activation and resulted in increased MED15 expression despite TGFß-receptor inhibition (Figure 4f), suggesting a TGFß independent PI3K/AKT signaling. In our previous study, we reported a significant increase in MED15 expression following TGFß3 treatment when cells were serum starved [12]. In this study, we treated cells in full growth medium to preserve high TGFß signaling for analyzing effects of TGFß inhibition by SB431542. Under these conditions, we did not observe changed MED15 levels in response to TGFß stimulation (Figure 4f), indicating that TGFß activity preserves but not highly up-regulates MED15 expression.

Due to their important roles during progression to CRPC, components of PI3K signaling currently are considered promising targets for the treatment of patients [38]. Therefore, several PI3K/AKT/mTOR inhibitors are currently in clinical trials and show promising benefits for patients [38]. However, predictive markers for the likelihood of responsiveness to these drugs are needed, and perhaps inhibitors of PI3K/AKT/mTOR signaling further downstream in the pathway might be more effective. These challenges are even more important considering the toxic side effects of these relative unselective pathway inhibitors [38]. As MED15 up-regulation correlates significantly with PI3K hyper-activation following ADT in tissues (Figure 3d) and is directly affected by modulating PI3K activity in cell lines (Figure 4b-4f), MED15 may serve as a predictive marker to select patients for the rational to use PI3K/AKT/mTOR inhibitors. Our previous results showing MED15 overexpression to correlate with worse clinical outcome [12], to affect proliferative activity of PCa cells and TGFß driven proliferation [12], provides evidence that MED15 drives oncogenic properties of advanced PCa and CRPC cells. Together with findings of this study, MED15 may serve as therapeutic target in the combination with TGFß and PI3K inhibitors to increase their efficiency and specify pathway inhibition. Further studies are needed to explore the functional role of MED15 during castration-resistance, and the molecular involvement in compensatory pathways enabling cells to survive and grow under androgen deprived conditions.

Our previous results showing MED15 overexpression to correlate with worse clinical outcome [12] provide evidence that MED15 drives oncogenic properties of advanced PCa and CRPC cells. Consistent with that, we show that *MED15* knockdown in LNCaP cells grown under androgen deprivation significantly reduces cell survival (Figure 5a-5c) which might rather be mediated by increased apoptosis than by altered cell cycle (Supplementary Figure 2a). Together with our tissue findings which show increased MED15 after ADT, this observation gives evidence that cells exhibiting strong MED15 expression are selected during androgen deprived conditions due to survival advantages. We show that reduction of MED15 leads to strong induction of apoptosis when androgens are removed, suggesting MED15 to serve as target for therapeutic intervention for CRPC. Inhibition of MED15 may prevent the selection of cells which are able to survive and grow despite low levels of androgens. Further studies are needed to explore the molecular mechanisms of apoptosis induced by *MED15* knockdown. In CRPC cells, resistance mechanisms include both AR dependent and AR independent signaling. We observed that MED15 associates with PI3K activity described as AR independent as well as AR interacting signaling. Interestingly, we found that *MED15* knockdown reduces protein expression of the AR while the AR co-regulator MED1 was not affected (Figure 5d). Decreased AR expression might lead to reduced AR mediated gene expression allowing cells to grow and survive under androgen deprivation. However, further experiments are needed to explore whether this might be an indirect effect of general reduced gene expression due to *MED15* knockdown mediated apoptosis of LNCaP cells observed under androgen deprivation (Figure 5b, 5c).

To recap, we demonstrate here that the expression of MED15 is elevated *in vivo* and *in vitro* after ADT and androgen-deprivation, respectively. MED15 correlates with high TGFß and PI3K pathway activity in prostate tissue, and modulating the activity of TGFß and PI3K in cell lines under androgen deprived conditions effects the expression of MED15. Based on our results, we suggest MED15 to be directly involved in adaptive signaling activated by androgen deprivation and may play a role in the progression to castration-resistance. Therefore, MED15 may serve as predictive marker for the use of drugs targeting TGFß and PI3K signaling, and as therapeutic target for CRPC.

## MATERIALS AND METHODS

This study was approved by the Internal Review Board of the University Hospital of Bonn in accordance with the Declaration of Helsinki.

### Cohorts

The cohort used in this study consists of total of 257 PCa tissue samples from patients who underwent palliative transurethral resection of the prostate (pTURP) at the University Hospital or the Clara Hospital in Basel, Switzerland as previously described [39]. Each sample comprises up to 3 cores. In more detail, the cohort is comprised of 102 hormone-naïve local recurrences and 10 hormone-naïve metastatic PCa samples, as well as 110 local recurrent castration-resistant PCa samples and 35 metastatic castration-resistant PCa samples from patients after undergoing androgen-deprivation therapy (ADT). PSMAD3 and pAKT staining was assessable for 227 and 236 PCa samples, respectively. For 29 patients, matched hormone-naïve local recurrent samples before androgen deprivation therapy (ADT) and local recurrent castration-resistant PCa samples after ADT (CRPC) were available. For most of these patients, 1 sample before ADT and 1 sample after ADT was available, while for 7 patients, 1 sample before ADT and 2 distinct samples from different time points after ADT were available (n=36).

### Immunohistochemistry

IHC was performed as described previously [12]. Briefly, staining was performed on sections of paraffin embedded tissues using Ventana XT immunostainer (Ventana, Tuscon, AZ). The following primary antibodies were used (dilution, clone, company): anti-MED15 rabbit polyclonal (1:50, 11566-1-AP, Proteintech, Chicago, IL), anti-p-SMAD3(S423+S425) rabbit monoclonal (1:50, EP823Y, Abcam, Cambridge, UK) and anti-p-AKT(Ser473) rabbit monoclonal (1:50, 736E11, Cell Signaling, Danvers, MA). The positive control tissue for MED15 was human prostate cancer, for pSMAD3 human liver cancer and for pAKT human lung cancer. As negative controls for MED15-, pSMAD3- and pAKT antibodies, same IHC protocol without adding primary antibody was performed and served as control for IHC selectivity. Quantitative analysis of MED15, p-SMAD3 and p-AKT expression was performed using Tissue Studio (Definiens Developer XD 2.0) as described previously [40]. Nuclear expression score (ES) of MED15 was defined as mean staining intensity per core. ES of pSMAD3 and pAKT was calculated as mean staining intensity of the cytoplasm and nucleus per core. For each sample, the average ES of available cores excluding outliers was analyzed. For statistical and correlation analysis, we used the full range ES for MED15, pSMAD3 and pAKT. A fold change expression > 1.1 after ADT relative to samples before ADT (=1) was defined as “increased expressed” after ADT. The median of the ES was used as cutoff value for dividing samples into high and low expression of MED15 (median=22), pAKT (median=11) and pSMAD3 (median=8.5).

### Cell treatments

All cell lines were purchased from the American Type Culture Collection (ATCC®, Manassas, VA) and were grown in a 5% CO_2_ incubator at 37 °C and 85% humidity. LNCaP cells were maintained in RPMI1640 medium (Biochrom, Berlin, Deutschland) containing 10% heat-inactivated fetal bovine serum (FBS, Sigma, St. Louis, MO) or charcoal stripped fetal bovine serum (CS FBS, Sigma, St. Louis, MO), 1% streptomycin-penicillin antibiotics (gibco®), 1% glutamine, 25mM HEPES buffer PAA and 1% NEAA (Thermo Scientific Fisher, Darmstadt, Germany). PC3 cells were maintained in RPMI1640 medium containing 10% heat-inactivated FBS, 1% streptomycin-penicillin antibiotics and 1% glutamine. VCaP cells were maintained in Dulbecco´s Modified Eagle´s Medium (DMEM) containing 10% heat-inactivated FBS or CS FBS, 1% streptomycin-penicillin antibiotics and 1% glutamine.

Cells were treated with epidermal growth factor (EGF, Immunotools, Friesoythe, Germany), transforming growth factor beta 3 (TGFß3, Immunotools, Friesoythe, Germany), LY294002 (Cell signaling, Danvers, MA), rapamycin (Merck Millipore, Nottingham, UK) and SB431542 (Tocris Bioscience, Bristol, UK) with stated doses and times. Prior to treatments with EGF, LNCaP cells were grown in medium containing 5% CS FBS for 72 hours. Prior to treatments with inhibitors, LNCaP and VCaP cells were grown in medium containing 10% CS FBS for 72 hours. LNCaP and VCaP cell treatments were performed in medium containing 5% (EGF) or 10% (LY294002, rapamycin and SB431542) CS FBS. Prior to treatments with TGFß or EGF, PC3 cells were serum starved with medium containing 2% FBS for 48 hours. PC3 cell treatments were performed in medium containing 2% (TGFß, EGF) or 10% (SB431542) FBS. Control cells for LY294002, rapamycin and SB431542 were treated with equal amounts of vehicle DMSO.

### Immunocytochemistry

For immunocytochemistry (ICC), LNCaP cells were grown on glass slides in medium containing 10% CS FBS or 10% FBS as control for 72 hours. Cells were fixed with paraformaldehyde (PFA) overnight and then washed with PBS. ICC was performed according to published protocol for cultured cell lines by Cell Signaling Technology (Danvers, MA) using an anti-MED15 rabbit polyclonal antibody (1:200, 11566-1-AP, Proteintech, Chicago, IL) and anti-MED1 mouse monoclonal antibody (1:100, H-7, Santa Cruz, Texas, US).

### Western blot

For the preparation of whole protein cell lysates, cell pellets were washed with ice-cold phosphate buffered saline (PBS) and re-suspended in an extraction buffer for 60 minutes. The lysates were then centrifuged for 30 minutes at 13.000 rpm at 4°C. The supernatant with whole protein lysate was harvested, and protein concentration was measured using bicinchoninic acid (BCA)- Protein Assay Kit (Thermo Scientific, Darmstadt, Germany). Thereafter, whole cell extracts were fractionated by SDS-PAGE and transferred to a polyvinylidene difluoride (PVDF) membrane using a transfer apparatus according to the manufacturer's protocols (Bio-Rad, München, Germany). After incubation with 5% nonfat milk in TBST (10 mM Tris, pH 8.0, 150 mM NaCl, 0.5% Tween 20) for 30 min, the membrane was incubated with anti-MED15 rabbit polyclonal (1:200, 11566-1-AP, Proteintech, Chicago, IL), anti-ß-ACTIN monoclonal antibody (1:5000, A1978, St.Louis, MO); anti-pAKT(Ser473) rabbit monoclobal (1:1000, D9E, Cell Signaling, Danvers, MA) and anti-AR mouse monoclonal (1:500, AR441, DAKO, Santa Clara, CA) primary antibodies at 4 °C overnight. Membranes were washed three times for 10 min with TBST and incubated with a 1:5000 dilution of horseradish peroxidase-conjugated anti-mouse or anti-rabbit antibodies for 1 h. Blots were washed with TBST three times and developed with the ECL system (GE Healthcare Life Science, Freiburg, Germany) according to the manufacturer's protocols.

### qRT-PCR

RNA was isolated using RNeasy Mini Kit (Qiagen, Germany) and reverse transcribed using an iScript cDNA synthesis kit, according to manufacturer's instructions (Biorad, Germany). PCR reactions with a Power SYBR Green kit (Thermo Fisher Scientific, Darmstadt, Germany) were performed according to the manufacturer's instructions using Light-Cycler 480 II (Roche, Mannheim, Germany). For each sample in a given experiment, technical duplicate reactions were performed using ßActin as house-keeping gene. Fold changes were calculated using the formula 2^ddCT. Primer pairs used for MED15 (Applied Biosystems, Darmstadt, Germany): MED15-F-5′-CAAGGCTTCCGTGATCATCT-3′; MED15 –R-5′-AGCAGACAGCAGTACAGACAGC -3′.

### SiRNA mediated *MED15* knockdown

For *MED15* knockdown, we used SMARTpool – ON - TARGETplus *MED15* siRNA (Thermo Scientific, Darmstadt, Germany) and as control, we used ON - TARGET plus nontargeting pool (Thermo Scientific, Darmstadt, Germany). LNCaP were transfected with 100 nmol/L siRNA using Screenfect A (Genaxxon Bioscience GmbH, Ulm, Germany).

### MTT cell viability assay

*MED15* knockdown was performed in 96-well plates and after 24 hours, medium was changed to charcoal stripped medium. After additional 72 hours, cells were analyzed for viability using the MTT assays according to the manufacturer's protocol (Roche, Mannheim, Germany). Each experiment was independently repeated three times in triplicates.

### Annexin V/PI apoptosis assay

*MED15* knockdown was performed in 6-well plates and after 24 hours, medium was changed to charcoal stripped medium. After additional 72 hours, cells were stained with Annexin V and PI, and evaluated for apoptosis by flow cytometry according to the manufacturer's protocol (eBioscience, San Diego, USA). Briefly, cells were washed twice with PBS and binding buffer, stained with 5μl of Annexin V–FITC and 2.5μl of PI in 1X binding buffer for 15 min at room temperature protected from light. Apoptotic cells were determined using FACSCanto II Cell Analyzer (BD Bioscience, Heidelberg, Germany). Analysis of apoptotic cells included both, early apoptotic (Annexin V-positive, PI-negative) and late apoptotic (Annexin V-positive and PI-positive) cells. Each experiment was independently repeated six times.

### Cell cycle analysis

For cell cycle analysis, we performed propidium iodide (PI) DNA staining followed by flow cytometric analysis. In more detail, 96 hours after siRNA transfection, cells were harvested and used for flow cytometric analysis. Cells were fixed in ice-cold 70% ethanol for 30 min at 4 °C, washed three times with ice-cold PBS and resuspended in 200µl DNA staining solution (PBS containing 50µg/ml PI (Sigma Aldrich, Steinheim, Germany) and 100µg/ml Ribonuclease A (Sigma Aldrich, Steinheim, Germany)). After 15min incubation in dark, cell cycle was analyzed using a LSR II Cell Analyzer (BD Bioscience, Heidelberg, Germany). FCS Express 5 Flow Cytometry software (DeNovoTM Software, Glendale, USA) was used for analyzing flow cytometric data.

## SUPPLEMENTARY MATERIALS FIGURES



## References

[1] A1 Jemal, Bray F, Center MM, Ferlay J, Ward E, Forman D (2011). Global cancer statistics. CA Cancer J Clin.

[2] Feldman BJ, Feldman D (2001). The development of androgen-independent prostate cancer. Nat Rev Cancer.

[3] Drake CG, Sharma P, Gerritsen W (2014). Metastatic castration-resistant prostate cancer: new therapies, novel combination strategies and implications for immunotherapy. Oncogene.

[4] X1 Yuan, Balk SP (2009). Mechanisms mediating androgen receptor reactivation after castration. Urol Oncol.

[5] BA1 Hellerstedt, Pienta KJ (2002). The current state of hormonal therapy for prostate cancer. CA Cancer J Clin.

[6] A1 Bjartell, Montironi R, Berney DM, Egevad L (2011). Tumour markers in prostate cancer II: diagnostic and prognostic cellular biomarkers. Acta Oncol.

[7] T1 Karantanos, Corn PG, Thompson TC (2013). Prostate cancer progression after androgen deprivation therapy: mechanisms of castrate resistance and novel therapeutic approaches. Oncogene.

[8] FM1 Sirotnak, She Y, Khokhar NZ, Hayes P, Gerald W, Scher HI (2004). Microarray analysis of prostate cancer progression to reduced androgen dependence: studies in unique models contrasts early and late molecular events. Mol Carcinog.

[9] IE1 Eder, Haag P, Basik M, Mousses S, Bektic J, Bartsch G, Klocker H (2003). Gene expression changes following androgen receptor elimination in LNCaP prostate cancer cells. Mol Carcinog.

[10] A1 Lunardi, Ala U, Epping MT, Salmena L, Clohessy JG, Webster KA, Wang G, Mazzucchelli R, Bianconi M, Stack EC, Lis R, Patnaik A, Cantley LC (2013). A co-clinical approach identifies mechanisms and potential therapies for androgen deprivation resistance in prostate cancer. Nat Genet.

[11] KJ1 Pienta, Bradley D (2006). Mechanisms underlying the development of androgen-independent prostate cancer. Clin Cancer Res.

[12] Shaikhibrahim Z, Menon R, Braun M, Offermann A, Queisser A, Boehm D, Vogel W, Rüenauver K, Ruiz C, Zellweger T, Svensson M, Andren O, Kristiansen G (2014). MED15, encoding a subunit of the mediator complex, is overexpressed at high frequency in castration-resistant prostate cancer. Int J Cancer.

[13] Lewis BA, Reinberg D (2003). The mediator coactivator complex: functional and physical roles in transcriptional regulation. J Cell Sci.

[14] Malik S, Roeder RG (2010). The metazoan Mediator co-activator complex as an integrative hub for transcriptional regulation. Nat Rev Genet.

[15] Schiano C, Casamassimi A, Rienzo M, de Nigris F, Sommese L, Napoli C (2014). Involvement of Mediator complex in malignancy. Biochim Biophys Acta.

[16] Syring I, Klümper N, Offermann A, Braun M, Deng M, Boehm D, Queisser A, von Mässenhausen A, Brägelmann J, Vogel W, Schmidt D, Majores M, Schindler A (2016). Comprehensive analysis of the transcriptional profile of the Mediator complex across human cancer types. Oncotarget.

[17] F1 Yang, Vought BW, Satterlee JS, Walker AK, ZY Jim Sun, Watts JL, DeBeaumont R, Saito RM, Hyberts SG, Yang S, Macol C, Iyer L, Tjian R (2006). An ARC/Mediator subunit required for SREBP control of cholesterol and lipid homeostasis. Nature.

[18] Kato Y, Habas R, Katsuyama Y, Näär AM, He X (2002). A component of the ARC/Mediator complex required for TGF beta/Nodal signalling. Nature.

[19] Kim S, Gross DS (2013). Mediator recruitment to heat shock genes requires dual Hsf1 activation domains and mediator tail subunits Med15 and Med16. J Biol Chem.

[20] Jones E, Pu H, Kyprianou N (2009). Targeting TGF-beta in prostate cancer: therapeutic possibilities during tumor progression. Expert Opin Ther Targets.

[21] Zhang F, Lee J, Lu S, Pettaway CA, Dong Z (2005). Blockade of transforming growth factor-beta signaling suppresses progression of androgen-independent human prostate cancer in nude mice. Clin Cancer Res.

[22] Walker L, Millena AC, Strong N, Khan SA (2013). Expression of TGFβ3 and its effects on migratory and invasive behavior of prostate cancer cells: involvement of PI3-kinase/AKT signaling pathway. Clin Exp Metastasis.

[23] Shin I, Bakin AV, Rodeck U, Brunet A, Arteaga CL (2001). Transforming growth factor beta enhances epithelial cell survival via Akt-dependent regulation of FKHRL1.Mol. Biol Cell.

[24] BS1 Taylor, Schultz N, Hieronymus H, Gopalan A, Xiao Y, Carver BS, Arora VK, Kaushik P, Cerami E, Reva B, Antipin Y, Mitsiades N, Landers T (2010). Integrative genomic profiling of human prostate cancer. Cancer Cell.

[25] Murillo H, Huang H, Schmidt LJ, Smith DI, Tindall DJ (2001). Role of PI3K signaling in survival and progression of LNCaP prostate cancer cells to the androgen refractory state. Endocrinology.

[26] Mulholland DJ, Dedhar S, Wu H, Nelson CC (2006). PTEN and GSK3beta: key regulators of progression to androgen-independent prostate cancer. Oncogene.

[27] Akhurst RJ, Hata A (2012). Targeting the TGFβ signalling pathway in disease. Nat Rev Drug Discov.

[28] Bitting RL, Armstrong AJ (2013). Targeting the PI3K/Akt/mTOR pathway in castration-resistant prostate cancer. Endocr Relat Cancer.

[29] Vijayvargia R, May MS, Fondell JD (2007). A coregulatory role for the mediator complex in prostate cancer cell proliferation and gene expression. Cancer Res.

[30] Liu G, Sprenger C, Wu PJ, Sun S, Uo T, Haugk K, Epilepsia KS, Plymate S (2015). MED1 mediates androgen receptor splice variant induced gene expression in the absence of ligand. Oncotarget.

[31] Jin F, Claessens F, Fondell JD (2012). Regulation of androgen receptor-dependent transcription by coactivator MED1 is mediated through a newly discovered noncanonical binding motif. J Biol Chem.

[32] Zhang L, Zhou F, ten Dijke P (2013). Signaling interplay between transforming growth factor-β receptor and PI3K/AKT pathways in cancer. Trends Biochem Sci.

[33] Craft N, Chhor C, Tran C, Belldegrun A, DeKernion J, Witte ON, Said J, Reiter RE, Sawyers CL (1999). Evidence for clonal outgrowth of androgen-independent prostate cancer cells from androgen-dependent tumors through a two-step process. Cancer Res.

[34] Conery AR, Cao Y, Thompson EA, Townsend CM, Ko TC, Luo K (2004). Akt interacts directly with Smad3 to regulate the sensitivity to TGF-beta induced apoptosis. Nat Cell Biol.

[35] Wilkes MC, Mitchell H, Penheiter SG, Doré JJ, Suzuki K, Edens M, Sharma DK, Pagano RE, Leof EB (2005). Transforming growth factor-beta activation of phosphatidylinositol 3-kinase is independent of Smad2 and Smad3 and regulates fibroblast responses via p21-activated kinase-2. Cancer Res.

[36] Karan D, Kelly DL, Rizzino A, Lin MF, Batra SK (2002). Expression profile of differentially-regulated genes during progression of androgen-independent growth in human prostate cancer cells. Carcinogenesis.

[37] Cunningham D, You Z (2015). *In vitro* and in vivo model systems used in prostate cancer research. J Biol Methods.

[38] Liu P, Cheng H, Roberts TM, Zhao JJ (2009). Targeting the phosphoinositide 3-kinase pathway in cancer. Nat Rev Drug Discov.

[39] Zellweger T, Stürm S, Rey S, Zlobec I, Gsponer JR, Rentsch CA, Terracciano LM, Bachmann A, Bubendorf L, Ruiz C (2013). Estrogen receptor β expression and androgen receptor phosphorylation correlate with a poor clinical outcome in hormone-naive prostate cancer and are elevated in castration-resistant disease. Endocr Relat Cancer.

[40] Braun M, Kirsten R, Rupp NJ, Moch H, Fend F, Wernert N, Kristiansen G, Perner S (2013). Quantification of protein expression in cells and cellular subcompartments on immunohistochemical sections using a computer supported image analysis system. Histol Histopathol.

[41] Zhang L, Zhou F, ten Dijke P (2013). Signaling interplay between transforming growth factor-β receptor and PI3K/AKT pathways in cancer. Trends Biochem Sci.

