# Supplementary material for: MED15 overexpression in prostate cancer arises during androgen deprivation therapy via PI3K/mTOR signaling
Source: Oncotarget. 2016 Dec 10;8(5):7964–76. doi: 10.18632/oncotarget.13860 (PMC5352374; doi:10.18632/oncotarget.13860)
Supplement: Supplementary file 1 [file oncotarget-08-7964-s001.pdf]

MED15 overexpression in prostate cancer arises during androgen deprivation therapy via PI3K/mTOR signaling

SUPPLEMENTARY FIGURES

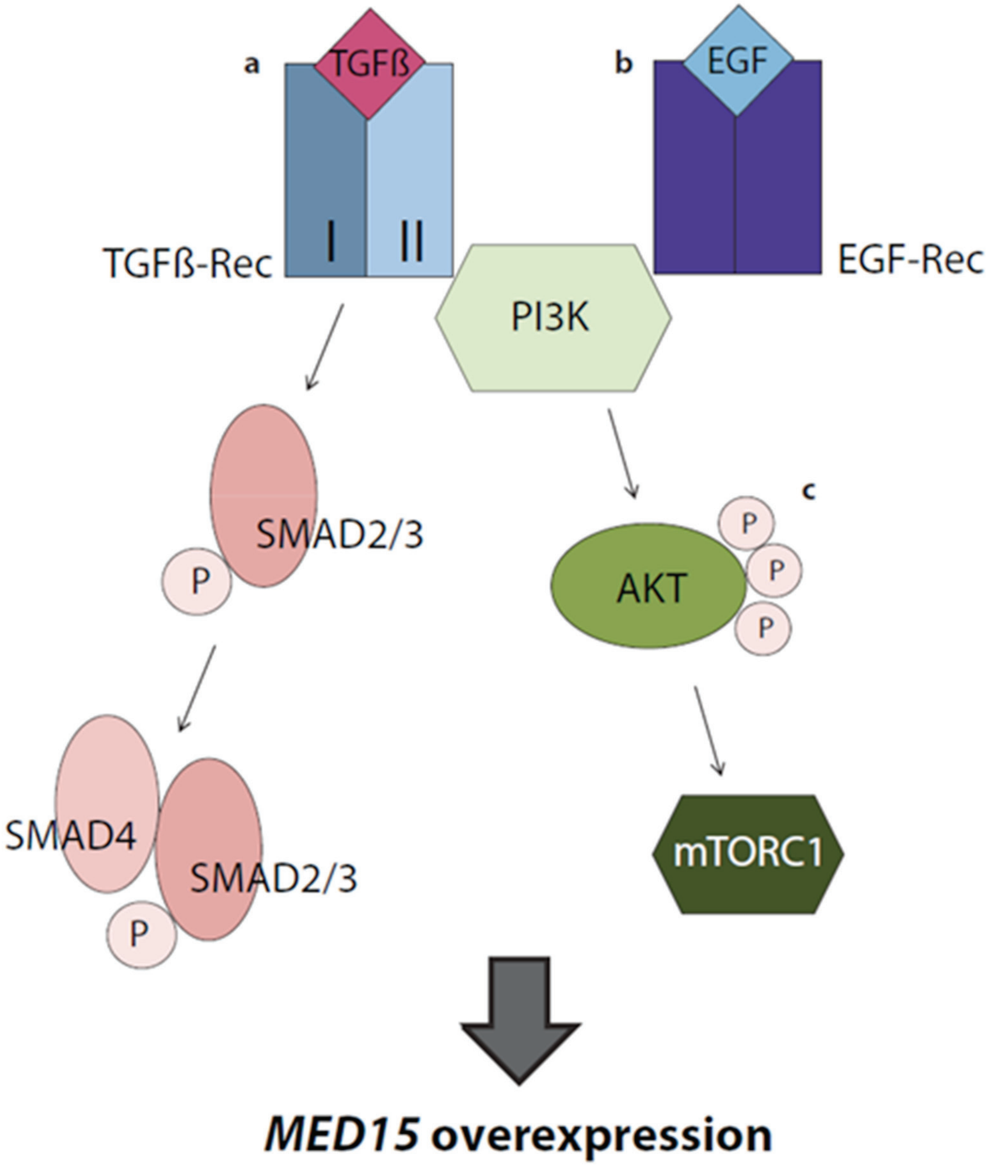

Supplementary Figure S1: Proposed working model. a. TGFß binding to the TGFß-receptor leads to SMAD signaling as well as PI3K signaling activation. **b.** EGF binding to EGF-receptor leads to PI3K signaling activation. **c.** Activation of the PI3K causes phosphorylation and activation of the downstream molecule AKT, followed by activation of mTOR. In our proposed working model, signaling activation of PI3K/AKT/mTOR and TGFß may lead to MED15 overexpression in PCa cells.

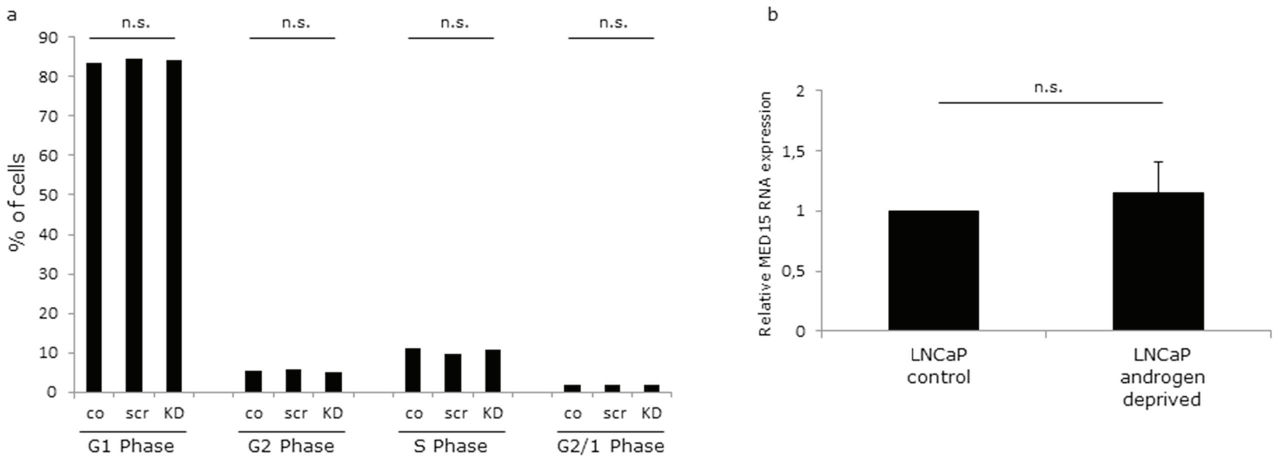

Supplementary Figure S2: a. Similar percentages of control cells and cells treated with scrambled or *MED15* specific siRNA in each cell cycle phase. **b.** qRT-PCR results show no significant different MED15 RNA expression level between cells growing in FBS or CS FBS.
